# Exploiting Global Structure Information to Improve Medical Image Segmentation

**DOI:** 10.3390/s21093249

**Published:** 2021-05-07

**Authors:** Jaemoon Hwang, Sangheum Hwang

**Affiliations:** 1 Department of Data Science, Seoul National University of Science and Technology, Seoul 01811, Korea; woans0105@ds.seoultech.ac.kr; 2 Department of Industrial & Information Systems Engineering, Seoul National University of Science and Technology, Seoul 01811, Korea; 3 Research Center for Electrical and Information Technology, Seoul National University of Science and Technology, Seoul 01811, Korea

**Keywords:** deep convolutional neural networks, medical image segmentation, structure information, domain robustness

## Abstract

In this paper, we propose a method to enhance the performance of segmentation models for medical images. The method is based on convolutional neural networks that learn the global structure information, which corresponds to anatomical structures in medical images. Specifically, the proposed method is designed to learn the global boundary structures via an autoencoder and constrain a segmentation network through a loss function. In this manner, the segmentation model performs the prediction in the learned anatomical feature space. Unlike previous studies that considered anatomical priors by using a pre-trained autoencoder to train segmentation networks, we propose a single-stage approach in which the segmentation network and autoencoder are jointly learned. To verify the effectiveness of the proposed method, the segmentation performance is evaluated in terms of both the overlap and distance metrics on the lung area and spinal cord segmentation tasks. The experimental results demonstrate that the proposed method can enhance not only the segmentation performance but also the robustness against domain shifts.

## 1. Introduction

Medical image segmentation is aimed at distinguishing the boundaries of lesions or organs in medical images acquired through X-ray, computed tomography (CT), magnetic resonance imaging (MRI), and other techniques. Segmentation results have been used to accomplish valuable clinical objectives in multiple practices, such as tumor detection to enable precise diagnosis and volume analysis to enable treatment planning [1].

Early studies on medical image segmentation adopted the methods such as edge detection, template matching, statistical shape models, and active contours [2]. The rapid development of deep learning has facilitated active research on image segmentation performed using convolutional neural networks (CNNs) [3]. A CNN is trained using images to produce accurate segmentation results by automatically learning hierarchical representations based on multiple stacked layers. Typically, a CNN-based segmentation model consists of an encoder that extracts features from an input image, and a decoder that restores the extracted features to the original image size through upsampling. U-Net [4], as a representative model having the encoder–decoder structure, effectively combines low- and high-level image features with skip-connections. Currently, U-Net and its variants have been widely used for many applications including medical image segmentation, and have outperformed other CNN-based architectures in terms of the segmentation performance [5,6,7,8].

Nevertheless, although CNN-based segmentation networks demonstrate a high prediction performance, these networks produce anatomically abnormal segmentation results in medical images in certain cases [9]. Compared to natural images, medical images are relatively well-standardized and contain anatomical structures that can be utilized as informative clues for segmentation. However, such information is not fully used in the learning process of CNN-based segmentation models. Considering these limitations, researchers have attempted to integrate the anatomical prior knowledge into the segmentation process in the medical imaging domain [10]. For example, contour information [11] or a low-dimensional representation of medical images from an autoencoder [12,13] has been used to learn anatomical priors.

Considering these aspects, this study is aimed at establishing a method of learning anatomical structures using an autoencoder such that the prediction of a segmentation network is performed in the learned anatomical feature space. This framework can help enhance the segmentation performance for the region of interest by reflecting the anatomical information in the learning process of the segmentation network. The proposed method differs from the existing approaches that use the anatomical features provided by pre-trained autoencoders [12,13]. Specifically, a single-stage method, in which the autoencoder is learned jointly with the segmentation network, is used to enhance the segmentation performance and training efficiency.

The proposed method was evaluated using the overlap measures including the intersection over union (IOU) and dice similarity coefficient (DSC), and distance measures such as the average contour distance (ACD) and average surface distance (ASD). To verify the effectiveness of the proposed method, we evaluated the performance of lung segmentation on chest X-rays in the widely used public benchmark datasets of the Japanese Society of Radiation Technology (JSRT) and Montgomery County (MC) [14,15,16] and the performance of the spinal cord segmentation on MRI pertaining to the ISMRM 2016 Spinal Cord Challenge dataset [17]. Comparative experiments were performed with the existing approaches that adopted pre-trained autoencoders, and the results demonstrated that the proposed method outperformed the comparison targets in the considered medical image segmentation tasks. Furthermore, the proposed method was noted to be robust to domain shifts, i.e., more accurate segmentation results were obtained on images from different domains such as gender, race, or imaging equipment manufacturers.

The remaining paper is organized as follows. The existing CNN-based segmentation networks that incorporate anatomical information into the learning process are introduced in Section 2. Section 3 describes the proposed method to effectively utilize the anatomical structure information during training. Section 4 presents the experimental settings including the medical image datasets and evaluation metrics as well as the experimental results. The concluding remarks are presented in Section 5.

## 2. Related Work

Fully convolutional networks (FCN) represent one of the early deep learning networks for semantic segmentation [18]. In an FCN, the fully connected layers that have been widely used in previous models for image classification such as VGG16 [19] and GoogleNet [20], are replaced by fully convolutional layers, which can take variable-sized images as inputs. The high-level features are combined with their low-level counterparts to obtain more accurate segmentation results. To further enhance the segmentation performance, an encoder–decoder structure that can learn how to upsample input features was proposed, and this architecture is currently widely implemented in various segmentation tasks [21,22,23,24]. In such an encoder–decoder architecture, each part provides certain functionalities. The encoder compresses and extracts the feature information to process the input data, and the decoder uses these compressed features (i.e., representations) to produce the segmentation outputs whose size is the same as that of the input images. For example, the deconvolution network [25] is composed of several decoder layers whose structure resembles the encoder.

Furthermore, U-Net is one of the most popular networks based on the encoder–decoder structure for segmentation tasks in the field of medical imaging [4]. This network combines the feature information extracted from the encoder with the outputs of the decoder layers to compensate for the information loss in the downsampling operations in the encoder layers. V-Net [26] is based on the encoder–decoder structure designed to solve the problem of prostate segmentation in 3D medical images. Specifically, residual-based learning is applied to train the V-Net, and a dice loss function is implemented to address the imbalance problem of the foreground and background in 3D medical images.

Many researchers have proposed novel architectures to enhance the segmentation performance; however, such architectures do not consider the common structural information of input images as the objective is usually to perform segmentation in natural images. However, in the case of medical images, the degree of standardization is usually relatively large. If a common structure exists in the full image, it is desirable to learn this structural information to obtain more robust and high performance segmentation networks.

In the medical imaging field, a key factor to be considered to enhance the segmentation performance is that a segmentation model must learn the prior knowledge regarding the anatomical structure, such as an organ’s shape or placement [12]. To this end, Chen et al. [11] used the contour information to train the model by employing the loss function with the contour label, and Dai et al. [27] performed adversarial training for a segmentation model to learn the overall structural information for heart segmentation. In this work, an auxiliary classification network was trained using class labels, and the output of the segmentation network was provided as an input to the trained classifier to update the model parameters in the segmentation network. Through this process, the shared characteristics of the class label and output of the segmentation network could be reflected in the segmentation network.

In addition, certain researchers used autoencoders to learn the low-dimensional features for anatomical structures. Oktay et al. [12] extracted the feature information by pre-training the autoencoder through segmentation labels. To apply the pre-trained feature information to a segmentation network, the outputs of the segmentation network (i.e., predictions) and segmentation labels were used as the inputs to the encoder part of the pre-trained autoencoder. The outputs of the encoder part (i.e., low-dimensional features) from the two inputs were compared with a loss function to ensure that the distributions of the output values were similar. Similar to [12], Tong et al. [13] adopted a strategy involving a pre-trained autoencoder trained using the ground-truth labels. This work introduced an additional loss function to minimize the difference between the two final outputs from the pre-trained autoencoder, specifically, the reconstructed output from the segmentation result and the ground-truth label.

Notably, the previous studies that reflected the anatomical structure information in CNN-based segmentation networks focused only on enhancing the segmentation performance and did not consider the robustness to domain shifts. In addition, although the approaches involving the autoencoder could effectively enhance the segmentation performance, two-stage methods were required to be used. Considering these aspects, this paper proposes a single-stage method in which the autoencoder and segmentation network are jointly trained to enhance not only the segmentation performance, but also the domain generalization capability.

## 3. Proposed Method

This section describes the proposed method, which includes a denoising convolutional autoencoder (DAE) and a segmentation network. The DAE is trained to learn the anatomical information by using segmentation labels (i.e., the ground-truth labels), and the segmentation network is trained to perform accurate segmentation. To reflect the anatomical information obtained by training the DAE in the segmentation network, the output of the last encoder layer of the segmentation network and corresponding output of the DAE are constrained through a loss function. Unlike previous studies that relied on a pre-training autoencoder, the proposed approach is a single-stage method in which the segmentation network and autoencoder are simultaneously trained. First, the details regarding the DAE employed in the proposed method are introduced, and subsequently, the overall framework of the proposed method is described.

### 3.1. Denoising Convolutional Autoencoder for Learning Anatomical Structures

The DAE is one of the dimensionality reduction methods based on deep neural networks, which can be used to extract the anatomical information in the data. In contrast to the conventional autoencoder, the DAE is trained to reconstruct the noise-added inputs to the original input data [28]. Gaussian, masking, and salt-and-pepper noises are commonly used to train the DAE. We considered the use of salt-and-pepper noise since the DAE is trained using binarized segmentation labels in the proposed framework.

**Figure 1:**
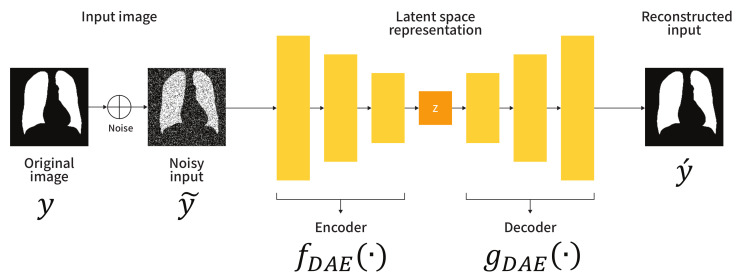
Architecture of the denoising convolutional autoencoder (DAE).

Figure 1 shows the schematic diagram of the adopted DAE. Let *y* be a segmentation label (i.e., a ground-truth) used as an input to the DAE. The encoder $f_{DAE}\left(\cdot \right)$ maps the corrupted input $\tilde{y}$ to a lower dimension, and the decoder $g_{DAE}\left(\cdot \right)$ uses these low-dimensional representations to produce the reconstructed input $\overset{´}{y}$. To generate the corrupted input $\tilde{y}$, randomly generated noise, e.g., salt-and-pepper noise, is added to the clean input *y*. The loss function $L_{DAE}$ to train the DAE can be expressed as:

(1) $$L_{DAE}=L\left(\overset{´}{y},y\right)$$

where the reconstructed input $\overset{´}{y}$ is computed as $\overset{´}{y}=g_{DAE}\left(z;\theta_{g_{DAE}}\right)$ and the compressed representation *z* is obtained as $z=f_{DAE}\left(\tilde{y};\theta_{f_{DAE}}\right)$. Here, $\theta_{f_{DAE}}$ and $\theta_{g_{DAE}}$ denote the learnable parameters of the encoder $f_{DAE}$ and decoder $g_{DAE}$, respectively. To train the DAE in the proposed framework, we employ the binary cross-entropy as a loss function *L*. As the training proceeds, the output *z* of the encoder contains the abstracted anatomical structure information learned by the data. In other words, the encoder performs an embedding that maps the input space to the anatomical structure space. Therefore, the feature space modeled by the encoder can be used to constrain the embedding space of the segmentation network.

### 3.2. Segmentation Network to Learn the Anatomical Structures

The goal of the segmentation network is to learn how to produce segmentation results from the anatomical structure space modeled by the DAE. To accomplish this goal, the output features from the segmentation encoder and DAE encoder should be tightly coupled. The proposed strategy is to apply the constraint by using a loss function to transfer the anatomical information being learned using the DAE to the segmentation network. In the proposed method, the U-Net architecture is used as a segmentation network.

**Figure 2:**
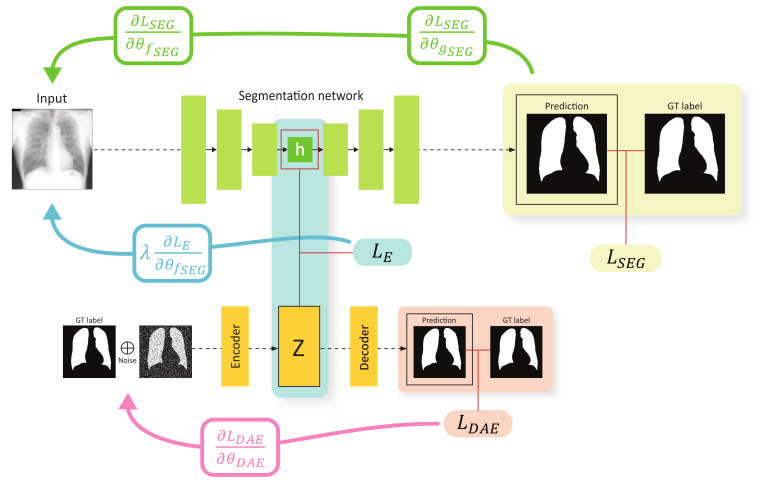
Training scheme of the proposed method. The green, blue, and red arrows show the gradient flow from $L_{SEG}$, $L_{E}$, and $L_{DAE}$, respectively. Note that the gradient from $L_{E}$ flows only through the encoder of the segmentation network.

The overall training scheme of the proposed method is illustrated in Figure 2. First, the segmentation network receives an image *x* as an input, and outputs a segmentation result $\overset{´}{x}$ whose spatial resolution is exactly the same with that of the input image. Specifically, the input image is embedded by the encoder $f_{SEG}$, resulting in the representation *h*, and then the decoder $g_{SEG}$ upsamples *h* through consecutive convolutional layers to produce a segmentation result. This network is trained using a loss function $L_{SEG}$ to perform pixel-level classification, which can be selected from various alternatives such as the binary cross-entropy, dice coefficient, etc. In this work, the binary cross-entropy loss for $L_{SEG}$ is adapted, which can be computed as:

(2) $$L_{SEG}=L\left(\overset{´}{x},y\right)$$

where $\overset{´}{x}=g_{SEG}\left(h;\theta_{g_{SEG}}\right)$ and $h=f_{SEG}\left(x;\theta_{f_{SEG}}\right)$. $\theta_{f_{SEG}}$ and $\theta_{g_{SEG}}$ denote the learnable parameters of the encoder $f_{SEG}$ and decoder $g_{SEG}$, respectively. The second component is the DAE. As explained in Section 3.1, the inputs of the DAE are the ground-truth labels to learn compact representations of the anatomical structures.

The key concept of the proposed method is to impose a constraint on the feature spaces constructed using the segmentation encoder $f_{SEG}$ and DAE encoder $f_{DAE}$ to ensure that the features for a specific data are similar. This constraint can be implemented by introducing an embedding loss function for *h* and *z* denoted as $L_{E}\left(h,z\right)$. Several loss functions can be used for $L_{E}$, e.g., mean squared error (MSE), mean absolute error (MAE), Kullback–Leibler divergence (KL), cosine loss, etc. We experimentally validated certain candidates for $L_{E}$ and finally selected the MSE.

In general, both the model parameters of $f_{DAE}$ and $f_{SEG}$ can be simultaneously trained using the embedding loss $L_{E}$. However, if the gradient update due to $L_{E}$ occurs in the segmentation network and DAE concurrently, the learning of the DAE may be affected, and the quality of the anatomical structure space constructed using the DAE may be degraded. Therefore, we intentionally design the gradient considering $L_{E}$ to update only the segmentation encoder $f_{SEG}$. In this framework, the segmentation encoder learns the mapping from the input space to the anatomical structure space, and the segmentation decoder is trained to perform segmentation with the features on the structure space. The proposed strategy for the gradient flow was empirically validated through experiments (refer to Section 4.3).

The total loss function $L_{Total}$ for the proposed method can be expressed as

(3) $$L_{Total}=L_{SEG}+L_{DAE}+\lambda L_{E}$$

where λ is a parameter that controls the weight of the embedding loss $L_{E}$. The optimal value is determined through the validation process. Note that the DAE in our proposed method is used as an auxiliary component during training, which helps the segmentation encoder learn better anatomical representations. Therefore, the DAE is discarded at an inference phase.

## 4. Experiments

This section describes the datasets and performance metrics considered to evaluate the proposed method. In addition, the results of experiments conducted on two segmentation tasks, lung segmentation in chest X-rays (CXR) and spinal cord segmentation in MRI, are presented, which demonstrate that the segmentation network trained using the proposed method exhibits a high performance and domain robustness. In addition, we present the results of an ablation study performed to validate the design choices in our proposed method.

### 4.1. Dataset

For lung segmentation, we used two public CXR datasets: JSRT [14] and MC [15]. JSRT is a dataset jointly created by the Japanese Society of Radiological Technology and the Japanese Radiological Society, and contains 247 the posterior-anterior (PA) CXR images. Among these images, 154 images have a pulmonary nodule, and the other 93 images are normal. All the images are sized 2048×2048 pixels and associated with the labeled annotations of other anatomical structures, including the lungs. The MC dataset is jointly populated by the National Library of Medicine and the Department of Health and Human Services in the U.S. This dataset consists of 138 PA CXR images; among these images, 80 images are normal and 58 correspond to tuberculosis patients. The images are sized 4020×4892 or 4892×4020 pixels.

For the spinal cord segmentation task with the MRI images, we used the dataset employed in the spinal cord gray matter challenge [17]. The dataset involves images collected from the following four sites: University College London (*site1*), Polytechnique Montreal (*site2*), University of Zurich (*site3*), and Vanderbilt University (*site4*). Specifically, the dataset consists of 80 MRI images corresponding to 20 cases from each site. The data from these sites exhibit individual visual characteristics mainly due to the imaging equipment from different vendors being used. Therefore, in the evaluation of the domain robustness of the segmentation networks, the images from each site were considered to correspond to a single domain.

**Figure 3:**
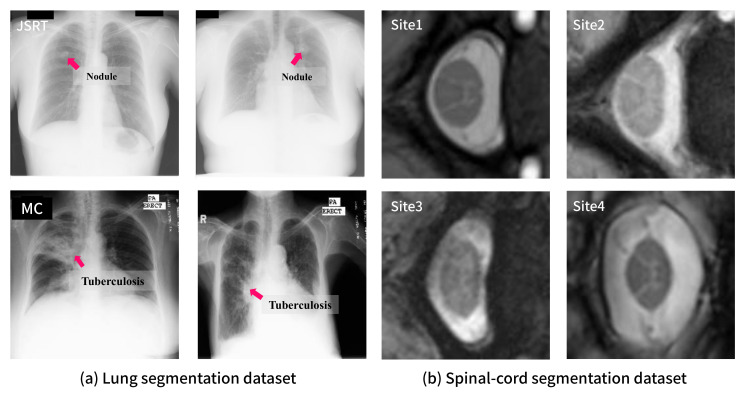
Examples of each dataset: (**a**) samples from JSRT (**top**) and MC (**bottom**) for lung segmentation with lesion annotations; (**b**) samples from four sites for spinal cord segmentation.

Figure 3 shows sample images of each dataset. From this figure, it can be observed that there exists a certain degree of distributional shift (i.e., domain difference) among the datasets. For example, the MC and JSRT datasets have considerably different visual features as can be observed through the annotations on the sample images in Figure 3. The JSRT dataset contains images from patients having lung nodules, which can be characterized as a small spot. In contrast, the MC dataset includes images from tuberculosis patients whose lesions are widely spread over the lung area. Such a domain shift is difficult to be resolved via image preprocessing (e.g., histogram equalization) or data augmentation (e.g., brightness and contrast adjustment) as observed in the following experiments.

### 4.2. Evaluation Metrics

To evaluate the segmentation performance, multiple performance metrics including overlap and distance measures were adopted.

- Intersection over union (IOU): the IOU is a measure of the degree of overlap between the region of the ground-truth and region predicted by the segmentation network. In Equation (4), *S* is the predicted region, and *G* is the ground-truth. The IOU can be defined as the ratio of the intersection and union between *G* and *S*:
  (4) $$\mathrm{IOU}=\left|\frac{G\;\cap \;S}{G\;\cup \;S}\right|=\frac{TP}{TP+FP+FN}$$
- Dice similarity coefficient (DSC): the DSC can be used to evaluate the overlap performance, similar to the IOU. This indicator also measures the degree of overlap between *S* and *G*, as indicated in Equation (5). Note that the DSC value is always greater than or equal to the IOU.
  (5) $$\mathrm{DSC}=\frac{2\left|\;G\;\cap \;S\;\right|}{\left|\;G\;\right|+\left|\;S\;\right|}=\frac{2TP}{2TP+FP+FN}$$
- Average contour distance (ACD) and average surface distance (ASD): the ACD and ASD indicate the extent of separation of the ground-truth and predicted region. Notably, the overlap measures such as the IOU and DSC do not consider whether the false positive pixels are near or far from the ground-truth. Let $s_{i}$, $i=1,\ldots ,n_{S}$ and $g_{i}$, $i=1,\ldots ,n_{G}$ represent the boundary pixels in *S* and *G*, respectively. $d\left(s_{i},G\right)=\min_{j}∥s_{i}-g_{j}∥$ indicates the minimum distance from $s_{i}$ on *S* to *G*. The ACD and ASD can be computed as follows:
  (6) $$\mathrm{ACD}\left(S,G\right)=\frac{1}{2}\left(\frac{\sum_{i}d\left(s_{i},G\right)}{n_{S}}+\frac{\sum_{j}d\left(g_{j},S\right)}{n_{G}}\right)$$
  (7) $$\mathrm{ASD}\left(S,G\right)=\frac{1}{n_{S}+n_{G}}\;\left(\sum_{i}d\left(s_{i},G\right)+\sum_{j}d\left(g_{j},S\right)\right)$$

### 4.3. Lung Segmentation Result

U-net [4] was utilized as a base segmentation network to perform comparative experiments involving existing frameworks such as the ACNN [12] and SRM [13] which consider the anatomical structures during training. Table 1 and Table 2 summarize the detailed architectures of the segmentation network and autoencoder for this experiment, respectively. As an activation function, the rectified linear unit (ReLU) was employed. Note that the feature maps *h* and *z* should be the same size to compute the embedding loss $L_{E}\left(h,z\right)$. The comparison targets, ACNN and SRM, were trained with the same architectures to enable a fair comparison.

In general, to apply the pre-trained anatomical information to the segmentation network, the ACNN performs lower-dimensional projections of both the segmentation predictions and ground-truths based on the pre-trained autoencoder, and computes the shape regularization loss between these projections. In this experiment, we adopted the binary cross-entropy and mean squared error as the segmentation loss and shape regularization loss, respectively, as in the original study. The weight of the shape regularization loss was set as 0.01 according to the validation process. The SRM [13] is a variant of the ACNN, which introduces an auxiliary loss function, specifically, the reconstruction loss, to ensure that the outputs from the projections obtained using the pre-trained autoencoder are similar. Therefore, the objective function in the SRM consists of three loss functions, specifically, the segmentation, shape regularization, and reconstruction losses. As in the original study, we used the dice loss as the segmentation and reconstruction loss, and the binary cross-entropy as the shape regularization loss. Through the validation process, the weights for the shape regularization and reconstruction loss were set as 0.01 and 0.001, respectively. For the proposed method, we set λ in Equation (3) as 1.0.

All the methods were trained using the Adam optimizer [29] with a learning rate of 0.0001 for 120 epochs. Histogram equalization was performed as a preprocessing step. For data augmentation, we performed brightness and contrast adjustment by setting the range from 0.8 to 1.2. The dataset was randomly split into a training, validation, and test dataset at a ratio of 65%, 15%, and 20%, respectively. To enable a rigorous evaluation, all the experiments were repeated five times, and the mean and standard deviation of the performance values were reported.

Table 3 presents the average and standard deviation of the performance values over five runs, corresponding to the proposed method and comparison targets. ↓ and ↑ indicate that lower and higher values are better, respectively. For each experiment, the best result is expressed in boldface. As baselines, the performances corresponding to the training of only a segmentation network with (U-Net) or without data augmentation (U-Net w/o aug) are reported in the table. The results on both the datasets indicate that data augmentation enhances the segmentation performance.

In addition, we observed that the methods in which the anatomical information was reflected during training, ACNN and SRM, outperformed the baselines, U-Net and U-Net w/o aug. Specifically, the ACNN and SRM exhibited an enhanced performance in terms of the distance metrics and all metrics in the case of the JSRT and MC datasets, respectively. Nevertheless, the proposed method outperformed all the methods in terms of all overlap and distance metrics on both the datasets. The comparison results indicated that in terms of the ASD, the proposed method exhibited an enhancement of 5.6% and 5.4% over the JSRT and MC datasets, respectively, against the second-best performing model SRM. This result demonstrated that the proposed method can help the segmentation network learn the global anatomical structure to be segmented by producing segmentation outputs from the anatomical feature space modeled by the autoencoder.

**Figure 4:**
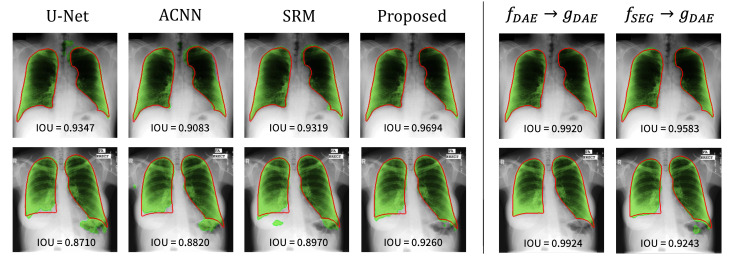
Visualization of the lung segmentation results on test samples from JSRT (**top**) and MC (**bottom**). The left part shows the results of each method, and the right part presents the results from $f_{DAE}\to g_{DAE}$ and $f_{SEG}\to g_{DAE}$ of the proposed method. The green color represents the segmentation results of each method, and the ground-truth is in red.

The visualization results are presented in Figure 4. The first and second rows show the segmentation results on the JSRT and MC dataset, respectively. The red solid line represents the ground-truth label, and the green area corresponds to the predicted result from the segmentation network. The left part shows the segmentation results of each method. The base U-Net tends to inaccurately predict the lung regions, and the reflection of the anatomical information in the network helps achieve better segmentation of the lung regions. Notably, the approach to use the anatomical information through the proposed strategy helped achieve the most accurate segmentation result.

To gain further insight into the proposed method, the reconstruction results from the trained DAE (i.e., $f_{DAE}\to g_{DAE}$) and segmentation results from the combination of segmentation encoder and DAE decoder (i.e., $f_{SEG}\to g_{DAE}$) are also depicted (see the right part of Figure 4). Here, we examined the reconstruction capability of DAE although it is not used during inference. The DAE reconstructs the input labels as well as expected since the reconstruction of binary lung masks is an easy task. The results from $f_{SEG}\to g_{DAE}$ are noteworthy: the features from $f_{SEG}$ can be successfully decoded by $g_{DAE}$. It implies that $f_{SEG}$ can embed an input image into the anatomical feature space modeled by the DAE, and thereby, $g_{DAE}$ can produce good segmentation results based on those features.

As described in Section 3.2, the proposed method is designed to control the gradient flows through the embedding loss function $L_{E}$ in Equation (3). The gradients from $L_{E}$ do not contribute to the DAE encoder, and thus, the DAE encoder is not affected when learning to build the anatomical structure features. Table 4 illustrates the effect of the proposed strategy on the JSRT dataset. Proposed-BI represents a method in which the gradient update from $L_{E}$ occurs in the segmentation and DAE encoder simultaneously, and Proposed-UN indicates the proposed strategy that only updates the segmentation encoder. From this experiment, we observed that Proposed-BI outperforms the baseline U-Net, which shows that constraining the feature space of the segmentation network by the autoencoder is effective to improve the segmentation performance. Moreover, the results of Proposed-UN demonstrate that preventing the gradient from $L_{E}$ from being propagated to the DAE further enhances the segmentation performance by encouraging the DAE to effectively learn the structural information.

### 4.4. Spinal Cord Segmentation Result

The spinal cord gray matter challenge dataset [17] was considered to evaluate the spinal cord segmentation performance of the proposed method. This dataset, which is composed of 3D MRI images, was cut cross-sectionally to allow the use of two-dimensional images in the experiment. Images without the ground-truth label were not used. Eventually, we considered 30, 113, 177, and 134 images from *site1*, *site2*, *site3*, and *site4*, respectively. Images from *site1* were not used for training due to the small number of images. The dataset corresponding to each site was split as follows: 65% for training, 15% for validation, and 20% for testing. All the images were center cropped at 128×128 pixels. The architectures of the segmentation network and autoencoder are similar to those in the lung segmentation experiment except for the number of layers and kernels: we used four times more kernels and removed one block in each encoder and decoder (i.e., $f_{4}$ and $g_{4}$ in Table 1 and Table 2) to build a better baseline.

The hyperparameters involved in each method were determined through a validation process: For the ACNN, the weight for the shape regularization loss was set as 0.001, and for the SRM, the weights for the shape regularization loss and reconstruction loss were set as 0.01 and 0.001, respectively. The weight λ for the proposed method was 1.0. All the methods were trained using the AdamP optimizer [30] for 120 epochs owing to the more stable training progress. The learning rate and weight decay parameter were set as 0.01 and 0.0001, respectively. To compare with stronger baselines, data augmentation strategies were adopted, including random adjustment of the brightness and contrast in the range of 0.6 to 1.4. The other experimental settings were the same as those in the lung segmentation experiment. To compare the performance, the mean and standard deviation of the performance metrics over five runs are reported in Table 5.

Similar to the results of the lung segmentation task, the ACNN and SRM outperformed the baseline U-Net, and the proposed method outperformed the comparison methods in terms of all metrics across the datasets from site2, site3, and site4. Notably, the distance metrics, ACD and ASD, were greatly improved, as in the previous experiment. For example, the ASD value of the proposed method on site2 was 0.372, 7.2% lower than the baseline and 2.6% lower than the second-best model ACNN.

**Figure 5:**
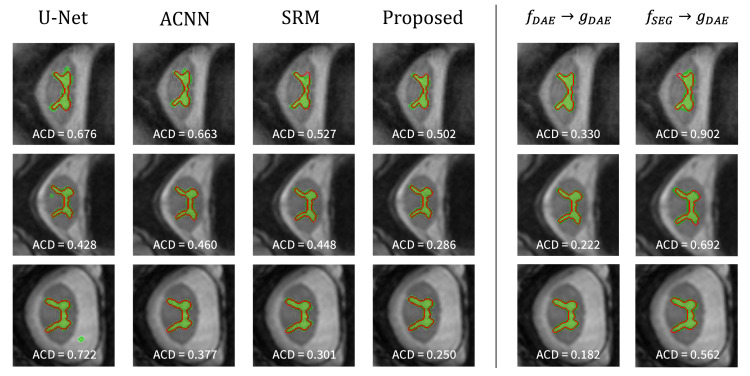
Visualization of the spinal cord segmentation results on test samples from *site2* (**top**), *site3* (**middle**), and *site4* (**bottom**). The left part shows the results of each method, and the right part presents the results from $f_{DAE}\to g_{DAE}$ and $f_{SEG}\to g_{DAE}$ of the proposed method. The green color represents the segmentation results of each method, and the ground-truth is in red.

The rows in Figure 5 show the predicted images from the segmentation methods for *site2*, *site3*, and *site4*, in order. The red solid line is the ground-truth label, and the green area represents the predicted result from the segmentation network. From the left part showing the comparison results, we can observe that the predictions from U-Net contain false positives located far from the ground-truth in several cases, although the other methods provide better segmentation results. Among these methods, the proposed method can realize more precise segmentation, especially in the case shown in the second row. These results demonstrate that the proposed method can more effectively learn the anatomical structure information than the comparison methods, resulting in better segmentation results. Similar to the lung segmentation task, the reconstruction results from $f_{DAE}\to g_{DAE}$ and segmentation results from $f_{SEG}\to g_{DAE}$ are presented (see the right part of Figure 5). From these visualization results, it is confirmed again that $f_{SEG}$ extracts anatomically informative features that can be easily decoded by $g_{DAE}$ trained to reconstruct the ground-truth labels.

### 4.5. Domain Robustness

Learning the anatomical structures in medical images can enhance several aspects of segmentation models, for instance, in the form of domain robustness. To demonstrate the domain robustness of the proposed method, we trained a segmentation network by using images from a single source (i.e., domain) and tested the trained model by using images from other sources. For example, the JSRT dataset was used for training, and the trained model’s performance was evaluated using the MC dataset in the case of the lung segmentation task. In general, if a network exhibits a high performance on datasets from unseen domains, the network is considered to be robust to domain shifts. We conducted similar experiments using the spinal cord dataset: images from each site, *site2*, *site3*, and *site4*, were used as the training images, and the segmentation performance was examined on images corresponding to other domains. Images from *site1* were utilized only for testing because the dataset for *site1* contains excessively few images to be used for training. The trained models in the previous experiments were used to examine their robustness to domain shifts.

The domain robustness (i.e., domain generalization) performance of the segmentation models in the lung segmentation task is summarized in Table 6. Two experimental settings were considered, JSRT→MC and MC→JSRT. In particular, JSRT→MC corresponds to the segmentation performances on the MC dataset for a model trained on the JSRT dataset. The last row presents the average performance over the two settings. The models trained using the proposed strategy exhibit superior performances in terms of both the overlap and distance measures, which indicates that the proposed method not only enhances the segmentation performance on the source domains, but also renders a segmentation model more robust to domain shifts. As can be seen in Figure 3, although the visual characteristics of the two datasets are considerably different, the experimental result highlights that the domain generalization performance of CNN-based segmentation models can be enhanced if we carefully design a training framework for the model to learn the anatomical structure information related to the given tasks.

**Figure 6:**
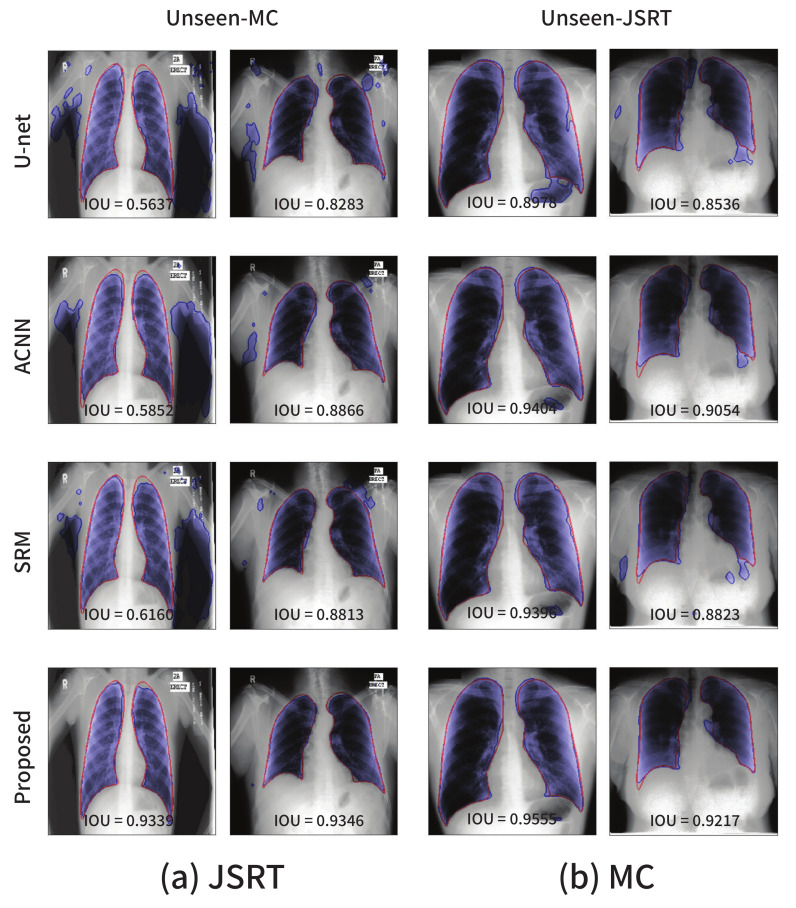
Segmentation results on the lung segmentation task obtained from the models trained using the (**a**) JSRT and (**b**) MC datasets, respectively. The blue color represents the segmentation results, and the ground-truth is in red.

The visualizations of several segmentation results are presented in Figure 6. The red solid line and blue shaded area represent the ground-truth and segmentation outputs, respectively. The existing approaches including the baseline U-Net are sensitive to even a small degree of domain shift, and this phenomenon cannot be simply resolved by applying data augmentation techniques such as random adjustments of the brightness and contrast.

For the spinal cord segmentation task, three experimental settings were considered: *site2*→others, *site3*→others, and *site4*→others. For models trained with each source domain, we evaluated the segmentation performance on the images from all other domains. The proposed method exhibited a higher generalization capability on unseen domains, as presented in Table 7. Except for the setting in which the source domain was *site3*, the proposed method achieved better segmentation results on other domains, especially in terms of the distance metrics. For example, when the source domain was *site4*, the ACD value averaged over other domains including *site1*, *site2*, and *site3* was improved by 14.8% compared to the second-best model SRM. According to the averaged performance over three experimental settings, the baseline U-Net, ACNN, and the proposed method showed a similar segmentation performance in terms of the overlap measures, although the distance performances of the proposed method were significantly improved. Specifically, the average ACD value for the proposed method was 0.667, corresponding to a 9.7% improvement over the baseline U-Net.

**Figure 7:**
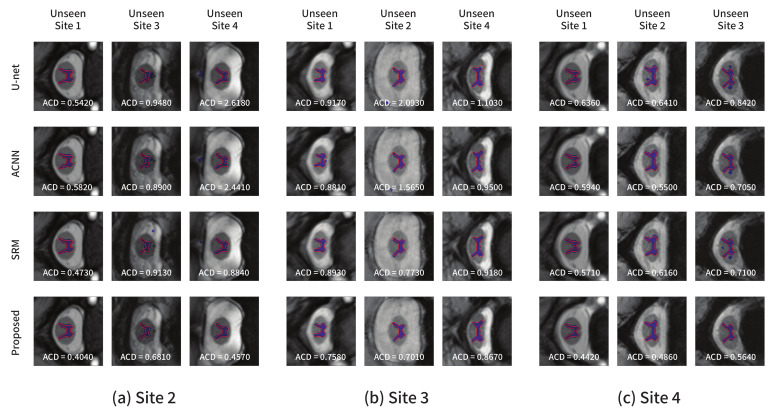
Segmentation results on the spinal cord segmentation task obtained from the models trained using the (**a**) *site2*, (**b**) *site3*, and (**c**) *site4* datasets, respectively. The blue color represents the segmentation results, and the ground-truth is in red.

Figure 7 shows the segmentation results obtained using each model from the test images in other domains not used for training. Figure 7a–c show the results from the models trained with *site2*, *site3*, and *site4* as the source domains, respectively. Similar conclusions as in the previous experiment could be derived. The comparison methods produced several false positive predictions, whereas the proposed method could accurately predict the target area. The quantitative and qualitative results indicated that valuable structural information such as a global shape or location in common across multiple domains can be learned using the proposed method.

## 5. Conclusions

In this paper, we propose a method to learn global anatomical structures in medical images by using a denoising convolutional autoencoder and constraining a segmentation network through a loss function such that the prediction of the segmentation model is performed in the learned anatomical feature space. Unlike previous studies in which anatomical priors are considered using a pre-trained autoencoder, we propose a single-stage approach in which the segmentation network and autoencoder are jointly learned. To demonstrate the advantages of the proposed method, extensive experiments were conducted on two medical image segmentation tasks: lung segmentation in CXRs and spinal cord segmentation in MRI images. The experimental results indicate that learning anatomical priors using the proposed method can help enhance the segmentation performance. In addition, to demonstrate the additional benefits of learning the anatomical structures, we investigated the domain robustness of the proposed method. The results indicate that the proposed method can enhance the robustness of segmentation networks against domain shifts. This domain robustness property will be particularly useful for other medical applications such as cranial implant design [31] or precise tooth segmentation [32] where understanding of anatomical structure is crucial to have reliable segmentation models. The findings highlight that the segmentation networks trained using the proposed method can effectively learn global anatomical structures commonly existing in medical images from various sources.

## Figures and Tables

**Table 1:** Detailed architecture of the segmentation network for lung segmentation, representing a kernel size, stride, the number of kernels, and size of output feature map of each layer.

		Kernel Size	Stride	Kernels	Feature Map
$f_{1}$	conv	3 × 3	1 × 1	16	256 × 256 × 16
	conv	3 × 3	1 × 1	16	256 × 256 × 16
	maxpool	2 × 2	2 × 2		128 × 128 × 16
$f_{2}$	conv	3 × 3	1 × 1	32	128 × 128 × 32
	conv	3 × 3	1 × 1	32	128 × 128 × 32
	maxpool	2 × 2	2 × 2		64 × 64 × 32
$f_{3}$	conv	3 × 3	1 × 1	64	64 × 64 × 64
	conv	3 × 3	1 × 1	64	64 × 64 × 64
	maxpool	2 × 2	2 × 2		32 × 32 × 64
$f_{4}$	conv	3 × 3	1 × 1	128	32 × 32 × 128
	conv	3 × 3	1 × 1	128	32 × 32 × 128
	maxpool	2 × 2	2 × 2		16 × 16 × 128
*h*	conv	3 × 3	1 × 1	256	16 × 16 × 256
	conv	3 × 3	1 × 1	256	16 × 16 × 256
$g_{4}$	deconv	2 × 2	2 × 2	128	32 × 32 × 128
	conv	3 × 3	1 × 1	128	32 × 32 × 128
	conv	3 × 3	1 × 1	128	32 × 32 × 128
$g_{3}$	deconv	2 × 2	2 × 2	64	64 × 64 × 64
	conv	3 × 3	1 × 1	64	64 × 64 × 64
	conv	3 × 3	1 × 1	64	64 × 64 × 64
$g_{2}$	deconv	2 × 2	2 × 2	32	128 × 128 × 32
	conv	3 × 3	1 × 1	32	128 × 128 × 32
	conv	3 × 3	1 × 1	32	128 × 128 × 32
$g_{1}$	deconv	2 × 2	2 × 2	16	256 × 256 × 16
	conv	3 × 3	1 × 1	16	256 × 256 × 16
	conv	3 × 3	1 × 1	16	256 × 256 × 16
output	conv	1 × 1	1 × 1	1	256 × 256 × 1

**Table 2:** Detailed architecture of the autoencoder network for lung segmentation, representing a kernel size, stride, the number of kernels, and size of output feature map of each layer.

		Kernel Size	Stride	Kernels	Feature Map
$f_{1}$	conv	3 × 3	1 × 1	16	256 × 256 × 16
	maxpool	2 × 2	2 × 2		128 × 128 × 16
$f_{2}$	conv	3 × 3	1 × 1	32	128 × 128 × 32
	maxpool	2 × 2	2 × 2		64 × 64 × 32
$f_{3}$	conv	3 × 3	1 × 1	64	64 × 64 × 64
	maxpool	2 × 2	2 × 2		32 × 32 × 64
$f_{4}$	conv	3 × 3	1 × 1	128	32 × 32 × 128
	maxpool	2 × 2	2 × 2		16 × 16 × 128
*z*	conv	3 × 3	1 × 1	256	16 × 16 × 256
$g_{4}$	deconv	2 × 2	2 × 2	256	32 × 32 × 256
	conv	3 × 3	1 × 1	128	32 × 32 × 128
$g_{3}$	deconv	2 × 2	2 × 2	128	64 × 64 × 128
	conv	3 × 3	1 × 1	64	64 × 64 × 64
$g_{2}$	deconv	2 × 2	2 × 2	64	128 × 128 × 64
	conv	3 × 3	1 × 1	32	128 × 128 × 32
$g_{1}$	deconv	2 × 2	2 × 2	32	256 × 256 × 32
	conv	3 × 3	1 × 1	16	256 × 256 × 16
output	conv	1 × 1	1 × 1	1	256 × 256 × 1

**Table 3:** Comparison of lung segmentation test performance. The means and standard deviations over five runs are reported. For each dataset, the best result is shown in boldface.

Dataset	Method	IOU(↑)	DSC(↑)	ACD(↓)	ASD(↓)
JSRT	U-Net w/o aug	0.950 ± 0.003	0.974 ± 0.002	1.376 ± 0.120	0.810 ± 0.023
	U-Net	0.955 ± 0.002	0.977 ± 0.001	1.116 ± 0.083	0.745 ± 0.017
	ACNN	0.955 ± 0.001	0.977 ± 0.000	1.036 ± 0.025	0.745 ± 0.007
	SRM	0.956 ± 0.001	0.977 ± 0.001	1.074 ± 0.023	0.732 ± 0.010
	Proposed	**0.959 ± 0.002**	**0.979 ± 0.001**	**0.936 ± 0.052**	**0.691 ± 0.013**
MC	U-Net w/o aug	0.940 ± 0.006	0.968 ± 0.004	1.547 ± 0.156	0.848 ± 0.039
	U-Net	0.950 ± 0.005	0.974 ± 0.003	1.212 ± 0.148	0.749 ± 0.032
	ACNN	0.953 ± 0.005	0.976 ± 0.003	1.069 ± 0.154	0.727 ± 0.044
	SRM	0.952 ± 0.003	0.975 ± 0.002	1.159 ± 0.139	0.726 ± 0.031
	Proposed	**0.956 ± 0.003**	**0.978 ± 0.002**	**1.032 ± 0.144**	**0.687 ± 0.026**

**Table 4:** Ablation study pertaining to the effect of controlling gradient flows. The best result is shown in boldface.

Method	IOU(↑)	DSC(↑)	ACD(↓)	ASD(↓)
U-Net	0.955 ± 0.002	0.977 ± 0.001	1.116 ± 0.083	0.745 ± 0.017
Proposed-BI	0.958 ± 0.002	0.978 ± 0.001	0.976 ± 0.054	0.705 ± 0.010
Proposed-UN	**0.959 ± 0.002**	**0.979 ± 0.001**	**0.936 ± 0.052**	**0.691 ± 0.013**

**Table 5:** Comparison of spinal cord segmentation test performance. The means and standard deviations over five runs are reported. For each dataset, the best result is shown in boldface.

Dataset	Method	IOU (↑)	DSC (↑)	ACD (↓)	ASD (↓)
*site2*	U-Net	0.793 ± 0.006	0.884 ± 0.004	0.585 ± 0.031	0.542 ± 0.015
	ACNN	0.792 ± 0.004	0.883 ± 0.003	0.592 ± 0.028	0.546 ± 0.013
	SRM	0.801 ± 0.006	0.889 ± 0.004	0.560 ± 0.036	0.518 ± 0.017
	Proposed	**0.805 ± 0.004**	**0.892 ± 0.002**	**0.536 ± 0.010**	**0.508 ± 0.009**
*site3*	U-Net	0.822 ± 0.007	0.900 ± 0.005	0.440 ± 0.033	0.401 ± 0.013
	ACNN	0.831 ± 0.004	0.905 ± 0.003	0.402 ± 0.009	0.382 ± 0.007
	SRM	0.828 ± 0.008	0.904 ± 0.004	0.398 ± 0.020	0.384 ± 0.018
	Proposed	**0.837 ± 0.01**	**0.909 ± 0.007**	**0.389 ± 0.034**	**0.372 ± 0.020**
*site4*	U-Net	0.853 ± 0.004	0.92 ± 0.002	0.423 ± 0.015	0.406 ± 0.009
	ACNN	0.856 ± 0.002	0.922 ± 0.001	0.413 ± 0.008	0.401 ± 0.005
	SRM	0.857 ± 0.003	0.923 ± 0.002	0.424 ± 0.030	0.398 ± 0.008
	Proposed	**0.858 ± 0.006**	**0.923 ± 0.004**	**0.408 ± 0.020**	**0.394 ± 0.015**

**Table 6:** Comparison of the domain robustness on lung segmentation. The best result on averaging over two settings is shown in boldface.

Setting	Method	IOU (↑)	DSC (↑)	ACD (↓)	ASD (↓)
JSRT→MC	U-Net	0.897 ± 0.008	0.943 ± 0.004	4.088 ± 1.053	1.377 ± 0.171
	ACNN	0.904 ± 0.006	0.947 ± 0.003	2.528 ± 0.500	1.112 ± 0.080
	SRM	0.902 ± 0.005	0.946 ± 0.002	3.481 ± 0.753	1.272 ± 0.111
	Proposed	0.924 ± 0.004	0.960 ± 0.002	2.101 ± 0.639	1.032 ± 0.095
MC→JSRT	U-Net	0.934 ± 0.001	0.966 ± 0.001	1.684 ± 0.055	0.987 ± 0.010
	ACNN	0.936 ± 0.001	0.967 ± 0.001	1.451 ± 0.049	0.945 ± 0.016
	SRM	0.935 ± 0.001	0.967 ± 0.001	1.580 ± 0.037	0.965 ± 0.011
	Proposed	0.938 ± 0.002	0.968 ± 0.001	1.388 ± 0.038	0.924 ± 0.012
Average	U-Net	0.916	0.955	2.886	1.182
	ACNN	0.920	0.957	1.990	1.029
	SRM	0.919	0.957	2.531	1.119
	Proposed	**0.931**	**0.964**	**1.745**	**0.978**

**Table 7:** Comparison of the domain robustness in the spinal cord segmentation task. The best result on averaging over three settings is shown in boldface.

Setting	Method	IOU (↑)	DSC (↑)	ACD (↓)	ASD (↓)
*site2*→others	U-Net	0.766 ± 0.005	0.863 ± 0.004	0.747 ± 0.033	0.579 ± 0.013
	ACNN	0.763 ± 0.006	0.861 ± 0.004	0.773 ± 0.054	0.591 ± 0.018
	SRM	0.758 ± 0.014	0.856 ± 0.012	0.655 ± 0.038	0.567 ± 0.017
	Proposed	0.768 ± 0.004	0.864 ± 0.003	0.654 ± 0.002	0.558 ± 0.006
*site3*→others	U-Net	0.747 ± 0.010	0.853 ± 0.007	0.685 ± 0.028	0.610 ± 0.016
	ACNN	0.741 ± 0.010	0.849 ± 0.007	0.697 ± 0.024	0.626 ± 0.018
	SRM	0.709 ± 0.020	0.824 ± 0.015	0.765 ± 0.059	0.667 ± 0.035
	Proposed	0.740 ± 0.012	0.847 ± 0.009	0.686 ± 0.029	0.615 ± 0.018
*site4*→others	U-Net	0.766 ± 0.004	0.864 ± 0.003	0.784 ± 0.050	0.583 ± 0.016
	ACNN	0.766 ± 0.002	0.864 ± 0.001	0.780 ± 0.033	0.585 ± 0.012
	SRM	0.767 ± 0.003	0.864 ± 0.001	0.775 ± 0.034	0.582 ± 0.010
	Proposed	0.767 ± 0.006	0.864 ± 0.006	0.660 ± 0.037	0.556 ± 0.012
Average	U-Net	**0.760**	**0.860**	0.739	0.591
	ACNN	0.757	0.858	0.750	0.601
	SRM	0.745	0.848	0.732	0.605
	Proposed	0.758	0.858	**0.667**	**0.576**

## Data Availability

All datasets used for this study are publicly available. Refer to the description of each dataset.
